# Bis[μ-1,1′-(ferrocene-1,1′-di­yl)bis(butane-1,3-dionato)]di-μ-methanol-diiron(II)

**DOI:** 10.1107/S1600536809016353

**Published:** 2009-05-07

**Authors:** Yan-Feng Xiao, Hong-Feng Li, Peng-Fei Yan, Guang-Ming Li

**Affiliations:** a Key Laboratory of Functional Inorganic Material Chemistry (Heilongjiang University), Ministry of Education, School of Chemistry and Materials Science, Heilongjiang University, 74 Xuefu Road, Nangang District, Harbin 150080, People’s Republic of China

## Abstract

The asymmetric unit of the title compound, [Fe_4_(C_9_H_8_O_2_)_4_(CH_3_OH)_2_], contains one half-mol­ecule located on a twofold rotational axis. In the mol­ecule, the two Fe^II^ ions bridged by two coordinating methanol mol­ecules are separated by 3.1286 (7) Å. Two crystallographically independent methanol mol­ecules are situated on a twofold rotational axis; all attached H atoms are rotationally disordered between two equal orientations.

## Related literature

For the similar cobalt and manganese complexes of the same beta-diketone, see: Yan *et al.* (2007 ▶).
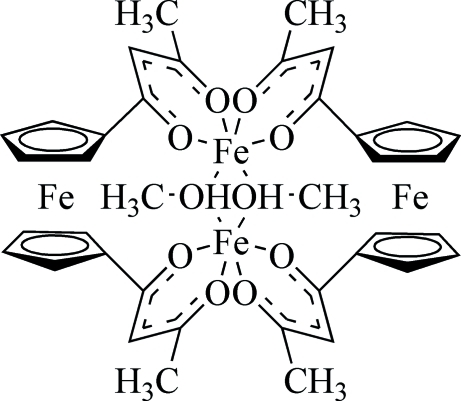


## Experimental

### 

#### Crystal data

[Fe_4_(C_9_H_8_O_2_)_4_(CH_4_O)_2_]
*M* *_r_* = 880.10
Orthorhombic, 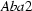

*a* = 14.599 (3) Å
*b* = 19.290 (4) Å
*c* = 12.955 (3) Å
*V* = 3648.5 (13) Å^3^
*Z* = 4
Mo *K*α radiation
μ = 1.62 mm^−1^
*T* = 291 K
0.25 × 0.22 × 0.20 mm

#### Data collection

Rigaku R-AXIS RAPID diffractometer
Absorption correction: multi-scan (*ABSCOR*; Higashi, 1995 ▶) *T*_min_ = 0.685, *T*_max_ = 0.737
16837 measured reflections
4079 independent reflections
3473 reflections with *I* > 2σ(*I*)
*R*_int_ = 0.042

#### Refinement

*R*[*F* ^2^ > 2σ(*F* ^2^)] = 0.031
*wR*(*F* ^2^) = 0.069
*S* = 1.05
4079 reflections
242 parameters
13 restraints
H-atom parameters constrained
Δρ_max_ = 0.24 e Å^−3^
Δρ_min_ = −0.28 e Å^−3^
Absolute structure: Flack (1983 ▶), 1899 Friedel pairs
Flack parameter: −0.001 (16)

### 

Data collection: *RAPID-AUTO* (Rigaku, 1998 ▶); cell refinement: *RAPID-AUTO*; data reduction: *CrystalStructure* (Rigaku/MSC, 2002 ▶); program(s) used to solve structure: *SHELXS97* (Sheldrick, 2008 ▶); program(s) used to refine structure: *SHELXL97* (Sheldrick, 2008 ▶); molecular graphics: *SHELXTL* (Sheldrick, 2008 ▶); software used to prepare material for publication: *SHELXL97*.

## Supplementary Material

Crystal structure: contains datablocks I, global. DOI: 10.1107/S1600536809016353/cv2543sup1.cif

Structure factors: contains datablocks I. DOI: 10.1107/S1600536809016353/cv2543Isup2.hkl

Additional supplementary materials:  crystallographic information; 3D view; checkCIF report

## Figures and Tables

**Fig. 1.**
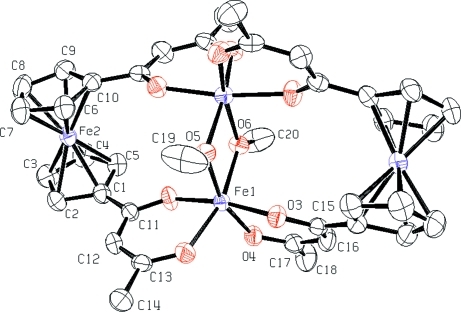
The molecular structure of (I) showing the atomic numbering and 66% probability displacement ellipsoids. Unlabelled atoms are related to labelled atoms by the symmetry code (-x, 2 - y, z). H atoms omitted for clarity.

## supplementary crystallographic information

### Comment

Recently, the cobalt and manganese complexes of the specific beta-diketone were published by Yan *et al.* (2007). Herewith we present the crystal structure of the title Fe^II^ complex with the same ligand, (I),

In (I) (Fig. 1), the tetradentate ferrocene-containing bis-beta-diketones ligand links Fe atoms into a complex through four O atoms of the two beta-diketone from two ligands. The two Fe^II^ ions and two 1,1'-bis(acetoacetyl)ferrocenes are bridged by two methanols molecules,  dividing this macrocyclic framework into two small cyclic subunits.The four atoms - C19, C20, O5 and O6 - of the two methanol molecules lie on a twofold axis.

### Experimental

The title complex was obtained by the treatment of iron(II) chloride tetrahydrate (0.5 mmol, 0.099 g) with 1,1'-bis(acetoacetyl)ferrocene (0.5 mmol, 0.177 g) in methanol. The first two reactants were stirred for 12 h. The resulting precipitates were filtered, washed with methanol several times and dried; The deep red crystals were grown by slow evaporation of methanol. Single crystals were obtained after several weeks. Analysis calculated for C_38_H_38_Fe_4_O_10_: C, 51.98; H, 4.36; found: C, 50.83; H, 4.02%.

### Refinement

All H atoms were placed in calculated positions (C—H = 0.93-0.97 Å, O—H = 0.82 Å) and treated as riding on their parent atoms, with *U*_iso_(H) = 1.2-1.5Ueq(C, O).

### Crystal data

**Table:**

[Fe_4_(C_9_H_8_O_2_)_4_(CH_4_O)_2_]	*F*(000) = 1808
*M**_r_* = 880.10	*D*_x_ = 1.602 Mg m^−^^3^
Orthorhombic, *A**b**a*2	Mo *K*α radiation, λ = 0.71073 Å
Hall symbol: A 2 -2ac	Cell parameters from 13899 reflections
*a* = 14.599 (3) Å	θ = 3.2–27.5°
*b* = 19.290 (4) Å	µ = 1.62 mm^−^^1^
*c* = 12.955 (3) Å	*T* = 291 K
*V* = 3648.5 (13) Å^3^	Block, colourless
*Z* = 4	0.25 × 0.22 × 0.20 mm

### Data collection

**Table:**

Rigaku R-AXIS RAPID diffractometer	4079 independent reflections
Radiation source: fine-focus sealed tube	3473 reflections with *I* > 2σ(*I*)
graphite	*R*_int_ = 0.042
ω scans	θ_max_ = 27.5°, θ_min_ = 3.1°
Absorption correction: multi-scan (*ABSCOR*; Higashi, 1995)	*h* = −18→18
*T*_min_ = 0.685, *T*_max_ = 0.737	*k* = −24→24
16837 measured reflections	*l* = −16→16

### Refinement

**Table:**

Refinement on *F*^2^	Hydrogen site location: inferred from neighbouring sites
Least-squares matrix: full	H-atom parameters constrained
*R*[*F*^2^ > 2σ(*F*^2^)] = 0.031	*w* = 1/[σ^2^(*F*_o_^2^) + (0.0338*P*)^2^ + 0.1021*P*] where *P* = (*F*_o_^2^ + 2*F*_c_^2^)/3
*wR*(*F*^2^) = 0.069	(Δ/σ)_max_ = 0.002
*S* = 1.05	Δρ_max_ = 0.24 e Å^−^^3^
4079 reflections	Δρ_min_ = −0.28 e Å^−^^3^
242 parameters	Extinction correction: *SHELXL97* (Sheldrick, 2008), Fc^*^=kFc[1+0.001xFc^2^λ^3^/sin(2θ)]^-1/4^
13 restraints	Extinction coefficient: 0.00077 (11)
Primary atom site location: structure-invariant direct methods	Absolute structure: Flack (1983), 1899 Friedel pairs
Secondary atom site location: difference Fourier map	Flack parameter: −0.001 (16)

### Special details

**Table:**

Geometry. All e.s.d.'s (except the e.s.d. in the dihedral angle between two l.s. planes) are estimated using the full covariance matrix. The cell e.s.d.'s are taken into account individually in the estimation of e.s.d.'s in distances, angles and torsion angles; correlations between e.s.d.'s in cell parameters are only used when they are defined by crystal symmetry. An approximate (isotropic) treatment of cell e.s.d.'s is used for estimating e.s.d.'s involving l.s. planes.
Refinement. Refinement of *F*^2^ against ALL reflections. The weighted *R*-factor *wR* and goodness of fit *S* are based on *F*^2^, conventional *R*-factors *R* are based on *F*, with *F* set to zero for negative *F*^2^. The threshold expression of *F*^2^ > σ(*F*^2^) is used only for calculating *R*-factors(gt) *etc*. and is not relevant to the choice of reflections for refinement. *R*-factors based on *F*^2^ are statistically about twice as large as those based on *F*, and *R*- factors based on ALL data will be even larger.

### Fractional atomic coordinates and isotropic or equivalent isotropic displacement parameters (Å^2^)

**Table:**

	*x*	*y*	*z*	*U*_iso_*/*U*_eq_	Occ. (<1)
C1	0.19291 (19)	0.81289 (13)	0.8190 (2)	0.0354 (7)	
C2	0.26358 (18)	0.76630 (12)	0.7841 (4)	0.0447 (7)	
H2	0.2613	0.7380	0.7213	0.054*	
C3	0.3373 (2)	0.76887 (17)	0.8546 (3)	0.0512 (9)	
H3	0.3945	0.7425	0.8498	0.061*	
C4	0.3135 (2)	0.81604 (17)	0.9337 (3)	0.0506 (8)	
H4	0.3518	0.8282	0.9931	0.061*	
C5	0.2264 (2)	0.84401 (15)	0.9114 (2)	0.0407 (7)	
H5	0.1938	0.8784	0.9533	0.049*	
C6	0.3017 (2)	0.93295 (15)	0.6728 (2)	0.0423 (7)	
H6	0.2494	0.9363	0.6257	0.051*	
C7	0.3795 (2)	0.88989 (17)	0.6594 (3)	0.0504 (8)	
H7	0.3905	0.8584	0.6013	0.060*	
C8	0.4386 (2)	0.90046 (18)	0.7442 (3)	0.0534 (9)	
H8	0.4974	0.8772	0.7553	0.064*	
C9	0.39808 (19)	0.94907 (15)	0.8111 (3)	0.0445 (8)	
H9	0.4241	0.9658	0.8763	0.053*	
C10	0.31223 (18)	0.96990 (14)	0.7674 (2)	0.0345 (7)	
C11	0.10328 (17)	0.83008 (12)	0.7745 (2)	0.0330 (7)	
C12	0.0715 (2)	0.79896 (15)	0.6841 (2)	0.0374 (7)	
H12	0.1085	0.7663	0.6518	0.045*	
C13	−0.0128 (2)	0.81460 (14)	0.6404 (2)	0.0360 (6)	
C14	−0.0418 (2)	0.77915 (17)	0.5423 (3)	0.0506 (8)	
H14A	−0.1028	0.7613	0.5503	0.076*	
H14B	−0.0005	0.7417	0.5276	0.076*	
H14C	−0.0406	0.8118	0.4864	0.076*	
C15	0.24318 (18)	1.01769 (12)	0.8099 (2)	0.0318 (7)	
C16	0.2553 (2)	1.04853 (15)	0.9063 (3)	0.0444 (7)	
H16	0.3070	1.0368	0.9445	0.053*	
C17	0.1943 (2)	1.09561 (14)	0.9477 (2)	0.0387 (7)	
C18	0.2130 (3)	1.12866 (18)	1.0513 (3)	0.0588 (10)	
H18A	0.2171	1.1780	1.0432	0.088*	
H18B	0.2696	1.1112	1.0785	0.088*	
H18C	0.1640	1.1178	1.0980	0.088*	
C19	0.0000	1.0000	0.5883 (5)	0.087 (2)	
H19A	−0.0225	1.0437	0.5636	0.130*	0.50
H19B	−0.0388	0.9634	0.5636	0.130*	0.50
H19C	0.0613	0.9929	0.5636	0.130*	0.50
C20	0.0000	1.0000	0.9899 (4)	0.078 (2)	
H20A	−0.0137	0.9542	1.0146	0.117*	0.50
H20B	−0.0455	1.0319	1.0146	0.117*	0.50
H20C	0.0592	1.0139	1.0146	0.117*	0.50
Fe1	−0.05321 (2)	0.929603 (15)	0.78857 (4)	0.02974 (10)	
Fe2	0.31370 (2)	0.865393 (17)	0.79403 (4)	0.03331 (10)	
O1	0.05773 (13)	0.87374 (10)	0.82598 (15)	0.0409 (5)	
O2	−0.06968 (14)	0.85889 (10)	0.67618 (17)	0.0406 (5)	
O3	0.17416 (13)	1.02878 (10)	0.75155 (16)	0.0367 (5)	
O4	0.11978 (14)	1.11480 (9)	0.90521 (16)	0.0382 (5)	
O5	0.0000	1.0000	0.6928 (2)	0.0346 (7)	
H20	−0.0100	1.0397	0.7126	0.052*	0.50
O6	0.0000	1.0000	0.8820 (2)	0.0319 (7)	
H21	0.0530	1.0026	0.8623	0.048*	0.50

### Atomic displacement parameters (Å^2^)

**Table:**

	*U*^11^	*U*^22^	*U*^33^	*U*^12^	*U*^13^	*U*^23^
C1	0.0409 (16)	0.0301 (12)	0.0351 (19)	0.0054 (11)	0.0030 (11)	0.0041 (12)
C2	0.0463 (15)	0.0309 (12)	0.0568 (18)	0.0085 (10)	0.004 (2)	−0.0019 (19)
C3	0.0468 (19)	0.0421 (17)	0.065 (2)	0.0151 (15)	−0.0060 (17)	0.0099 (17)
C4	0.055 (2)	0.059 (2)	0.0379 (18)	0.0072 (16)	−0.0096 (15)	0.0118 (16)
C5	0.0437 (18)	0.0459 (17)	0.0327 (15)	0.0087 (13)	−0.0019 (13)	0.0055 (14)
C6	0.0486 (19)	0.0465 (17)	0.0320 (16)	0.0059 (13)	0.0039 (14)	0.0056 (14)
C7	0.056 (2)	0.0577 (19)	0.0376 (17)	0.0114 (16)	0.0106 (15)	−0.0005 (16)
C8	0.0356 (17)	0.058 (2)	0.066 (2)	0.0113 (15)	0.0113 (16)	0.0046 (18)
C9	0.0336 (14)	0.0454 (15)	0.055 (3)	0.0009 (12)	−0.0060 (14)	−0.0036 (16)
C10	0.0340 (14)	0.0336 (13)	0.036 (2)	0.0000 (10)	−0.0001 (12)	0.0033 (12)
C11	0.0377 (14)	0.0269 (11)	0.0345 (18)	−0.0003 (10)	0.0080 (13)	−0.0012 (13)
C12	0.0381 (16)	0.0370 (15)	0.0371 (16)	0.0054 (12)	0.0066 (13)	−0.0091 (13)
C13	0.0421 (17)	0.0307 (13)	0.0352 (15)	−0.0099 (12)	0.0066 (13)	−0.0059 (12)
C14	0.049 (2)	0.055 (2)	0.0480 (19)	−0.0067 (15)	0.0030 (15)	−0.0194 (17)
C15	0.0344 (14)	0.0265 (11)	0.0347 (19)	−0.0048 (9)	−0.0053 (12)	0.0027 (12)
C16	0.0420 (17)	0.0434 (15)	0.0479 (19)	0.0061 (13)	−0.0190 (15)	−0.0071 (16)
C17	0.0453 (18)	0.0296 (14)	0.0412 (17)	−0.0037 (12)	−0.0112 (14)	−0.0044 (13)
C18	0.066 (2)	0.061 (2)	0.0492 (19)	0.0117 (16)	−0.023 (2)	−0.0177 (18)
C19	0.146 (7)	0.085 (4)	0.030 (3)	−0.044 (4)	0.000	0.000
C20	0.103 (5)	0.102 (5)	0.028 (3)	−0.047 (4)	0.000	0.000
Fe1	0.03050 (18)	0.02938 (16)	0.02933 (18)	−0.00004 (12)	0.0040 (2)	−0.0034 (2)
Fe2	0.03464 (19)	0.03372 (17)	0.03157 (19)	0.00768 (13)	−0.0017 (3)	−0.0015 (2)
O1	0.0402 (11)	0.0450 (11)	0.0374 (11)	0.0151 (9)	−0.0024 (8)	−0.0119 (9)
O2	0.0365 (12)	0.0411 (11)	0.0442 (12)	0.0003 (9)	−0.0024 (10)	−0.0119 (10)
O3	0.0345 (11)	0.0389 (10)	0.0366 (10)	0.0042 (8)	−0.0050 (8)	−0.0024 (9)
O4	0.0397 (12)	0.0296 (10)	0.0452 (12)	0.0028 (9)	−0.0111 (10)	−0.0053 (9)
O5	0.038 (2)	0.0422 (18)	0.0235 (15)	−0.0045 (14)	0.000	0.000
O6	0.042 (2)	0.0326 (16)	0.0215 (14)	−0.0051 (13)	0.000	0.000

### Geometric parameters (Å, °)

**Table:**

C1—C5	1.426 (4)	C13—O2	1.279 (3)
C1—C2	1.441 (4)	C13—C14	1.503 (4)
C1—C11	1.468 (4)	C14—H14A	0.9600
C1—Fe2	2.059 (3)	C14—H14B	0.9600
C2—C3	1.412 (5)	C14—H14C	0.9600
C2—Fe2	2.051 (3)	C15—O3	1.278 (3)
C2—H2	0.9800	C15—C16	1.394 (4)
C3—C4	1.413 (5)	C16—C17	1.381 (4)
C3—Fe2	2.050 (3)	C16—H16	0.9300
C3—H3	0.9800	C17—O4	1.274 (3)
C4—C5	1.412 (4)	C17—C18	1.511 (4)
C4—Fe2	2.044 (3)	C18—H18A	0.9600
C4—H4	0.9800	C18—H18B	0.9600
C5—Fe2	2.027 (3)	C18—H18C	0.9600
C5—H5	0.9800	C19—O5	1.355 (7)
C6—C7	1.418 (4)	C19—H19A	0.9600
C6—C10	1.426 (4)	C19—H19B	0.9600
C6—Fe2	2.048 (3)	C19—H19C	0.9600
C6—H6	0.9800	C20—O6	1.398 (6)
C7—C8	1.412 (5)	C20—H20A	0.9600
C7—Fe2	2.047 (3)	C20—H20B	0.9600
C7—H7	0.9800	C20—H20C	0.9600
C8—C9	1.407 (4)	Fe1—O6	1.9783 (17)
C8—Fe2	2.049 (3)	Fe1—O4^i^	1.991 (2)
C8—H8	0.9800	Fe1—O5	1.9964 (18)
C9—C10	1.433 (4)	Fe1—O3^i^	1.9982 (19)
C9—Fe2	2.043 (3)	Fe1—O1	2.0048 (19)
C9—H9	0.9800	Fe1—O2	2.010 (2)
C10—C15	1.473 (4)	O3—Fe1^i^	1.9982 (19)
C10—Fe2	2.045 (3)	O4—Fe1^i^	1.991 (2)
C11—O1	1.263 (3)	O5—Fe1^i^	1.9964 (18)
C11—C12	1.395 (4)	O5—H20	0.8211
C12—C13	1.389 (4)	O6—Fe1^i^	1.9783 (17)
C12—H12	0.9300	O6—H21	0.8158
			
C5—C1—C2	106.3 (3)	H18B—C18—H18C	109.5
C5—C1—C11	122.7 (2)	O5—C19—H19A	109.5
C2—C1—C11	131.0 (3)	O5—C19—H19B	109.5
C5—C1—Fe2	68.37 (17)	H19A—C19—H19B	109.5
C2—C1—Fe2	69.16 (15)	O5—C19—H19C	109.5
C11—C1—Fe2	126.20 (18)	H19A—C19—H19C	109.5
C3—C2—C1	108.7 (3)	H19B—C19—H19C	109.5
C3—C2—Fe2	69.82 (18)	O6—C20—H20A	109.5
C1—C2—Fe2	69.79 (15)	O6—C20—H20B	109.5
C3—C2—H2	125.6	H20A—C20—H20B	109.5
C1—C2—H2	125.6	O6—C20—H20C	109.5
Fe2—C2—H2	125.6	H20A—C20—H20C	109.5
C2—C3—C4	107.7 (3)	H20B—C20—H20C	109.5
C2—C3—Fe2	69.89 (16)	O6—Fe1—O4^i^	91.29 (9)
C4—C3—Fe2	69.60 (18)	O6—Fe1—O5	76.14 (7)
C2—C3—H3	126.1	O4^i^—Fe1—O5	162.62 (7)
C4—C3—H3	126.1	O6—Fe1—O3^i^	102.60 (6)
Fe2—C3—H3	126.1	O4^i^—Fe1—O3^i^	85.61 (8)
C5—C4—C3	108.6 (3)	O5—Fe1—O3^i^	85.50 (6)
C5—C4—Fe2	69.05 (18)	O6—Fe1—O1	84.48 (6)
C3—C4—Fe2	70.0 (2)	O4^i^—Fe1—O1	88.83 (9)
C5—C4—H4	125.7	O5—Fe1—O1	101.62 (6)
C3—C4—H4	125.7	O3^i^—Fe1—O1	171.08 (8)
Fe2—C4—H4	125.7	O6—Fe1—O2	163.02 (7)
C4—C5—C1	108.6 (3)	O4^i^—Fe1—O2	101.51 (8)
C4—C5—Fe2	70.37 (19)	O5—Fe1—O2	93.34 (9)
C1—C5—Fe2	70.80 (17)	O3^i^—Fe1—O2	89.60 (8)
C4—C5—H5	125.7	O1—Fe1—O2	84.66 (8)
C1—C5—H5	125.7	C5—Fe2—C9	117.30 (13)
Fe2—C5—H5	125.7	C5—Fe2—C4	40.58 (12)
C7—C6—C10	108.2 (3)	C9—Fe2—C4	105.82 (14)
C7—C6—Fe2	69.68 (18)	C5—Fe2—C10	108.69 (11)
C10—C6—Fe2	69.52 (17)	C9—Fe2—C10	41.04 (11)
C7—C6—H6	125.9	C4—Fe2—C10	127.46 (13)
C10—C6—H6	125.9	C5—Fe2—C7	169.03 (13)
Fe2—C6—H6	125.9	C9—Fe2—C7	68.09 (14)
C8—C7—C6	108.0 (3)	C4—Fe2—C7	149.56 (14)
C8—C7—Fe2	69.91 (19)	C10—Fe2—C7	68.51 (12)
C6—C7—Fe2	69.78 (18)	C5—Fe2—C6	130.60 (12)
C8—C7—H7	126.0	C9—Fe2—C6	68.41 (14)
C6—C7—H7	126.0	C4—Fe2—C6	167.07 (13)
Fe2—C7—H7	126.0	C10—Fe2—C6	40.77 (12)
C9—C8—C7	108.6 (3)	C7—Fe2—C6	40.54 (12)
C9—C8—Fe2	69.65 (17)	C5—Fe2—C8	149.67 (14)
C7—C8—Fe2	69.76 (19)	C9—Fe2—C8	40.23 (13)
C9—C8—H8	125.7	C4—Fe2—C8	115.68 (15)
C7—C8—H8	125.7	C10—Fe2—C8	68.34 (12)
Fe2—C8—H8	125.7	C7—Fe2—C8	40.33 (14)
C8—C9—C10	108.1 (3)	C6—Fe2—C8	67.97 (14)
C8—C9—Fe2	70.11 (18)	C5—Fe2—C3	68.49 (13)
C10—C9—Fe2	69.58 (15)	C9—Fe2—C3	125.10 (14)
C8—C9—H9	125.9	C4—Fe2—C3	40.38 (14)
C10—C9—H9	125.9	C10—Fe2—C3	164.08 (13)
Fe2—C9—H9	125.9	C7—Fe2—C3	117.22 (14)
C6—C10—C9	107.1 (3)	C6—Fe2—C3	152.39 (14)
C6—C10—C15	124.1 (3)	C8—Fe2—C3	105.73 (14)
C9—C10—C15	128.8 (3)	C5—Fe2—C2	68.45 (15)
C6—C10—Fe2	69.71 (16)	C9—Fe2—C2	163.45 (11)
C9—C10—Fe2	69.37 (16)	C4—Fe2—C2	67.72 (17)
C15—C10—Fe2	124.13 (18)	C10—Fe2—C2	154.74 (12)
O1—C11—C12	123.7 (2)	C7—Fe2—C2	109.24 (16)
O1—C11—C1	114.4 (2)	C6—Fe2—C2	120.98 (16)
C12—C11—C1	121.9 (2)	C8—Fe2—C2	127.27 (13)
C13—C12—C11	123.0 (2)	C3—Fe2—C2	40.29 (14)
C13—C12—H12	118.5	C5—Fe2—C1	40.83 (11)
C11—C12—H12	118.5	C9—Fe2—C1	152.63 (12)
O2—C13—C12	124.9 (3)	C4—Fe2—C1	68.34 (12)
O2—C13—C14	115.3 (3)	C10—Fe2—C1	120.15 (10)
C12—C13—C14	119.7 (3)	C7—Fe2—C1	130.52 (13)
C13—C14—H14A	109.5	C6—Fe2—C1	111.11 (12)
C13—C14—H14B	109.5	C8—Fe2—C1	166.90 (13)
H14A—C14—H14B	109.5	C3—Fe2—C1	68.70 (12)
C13—C14—H14C	109.5	C2—Fe2—C1	41.05 (10)
H14A—C14—H14C	109.5	C11—O1—Fe1	131.01 (19)
H14B—C14—H14C	109.5	C13—O2—Fe1	129.69 (19)
O3—C15—C16	124.0 (3)	C15—O3—Fe1^i^	128.48 (19)
O3—C15—C10	115.0 (2)	C17—O4—Fe1^i^	128.29 (18)
C16—C15—C10	121.0 (3)	C19—O5—Fe1^i^	128.41 (7)
C17—C16—C15	123.1 (3)	C19—O5—Fe1	128.41 (7)
C17—C16—H16	118.4	Fe1^i^—O5—Fe1	103.19 (13)
C15—C16—H16	118.4	C19—O5—H20	108.2
O4—C17—C16	125.1 (3)	Fe1^i^—O5—H20	40.6
O4—C17—C18	114.5 (3)	Fe1—O5—H20	111.8
C16—C17—C18	120.4 (3)	C20—O6—Fe1	127.74 (6)
C17—C18—H18A	109.5	C20—O6—Fe1^i^	127.74 (6)
C17—C18—H18B	109.5	Fe1—O6—Fe1^i^	104.52 (13)
H18A—C18—H18B	109.5	C20—O6—H21	108.2
C17—C18—H18C	109.5	Fe1—O6—H21	102.9
H18A—C18—H18C	109.5	Fe1^i^—O6—H21	52.7

Symmetry codes: (i) −*x*, −*y*+2, *z*.

### Hydrogen-bond geometry (Å, °)

**Table:**

*D*—H···*A*	*D*—H	H···*A*	*D*···*A*	*D*—H···*A*
C20—H20A···O4^i^	0.96	2.49	3.028 (3)	116
C20—H20C···O4	0.96	2.57	3.028 (3)	110
C19—H19B···O2	0.96	2.53	3.121 (3)	120
C3—H3···O4^ii^	0.98	2.57	3.107 (4)	114
O6—H21···O4	0.82	2.44	2.8377 (19)	111
O6—H21···O3	0.82	2.33	3.103 (3)	158
O5—H20···O1^i^	0.82	2.33	3.101 (3)	157
O5—H20···O2^i^	0.82	2.32	2.914 (2)	129

Symmetry codes: (i) −*x*, −*y*+2, *z*; (ii) −*x*+1/2, *y*−1/2, *z*.
